# Glucocorticoids on bone remodeling in systemic lupus erythematosus mice

**DOI:** 10.1038/s41390-025-03861-0

**Published:** 2025-01-24

**Authors:** Sheng Hao, Yuyun Zhang, Xiaowei Tong, Fangkai Ding, Runjie Wang, Jing Zhang, Dan Feng, Xiaoling Niu, Wenyan Huang

**Affiliations:** Department of Nephrology, Rheumatology and Immunology, Shanghai Children’s Hospital, School of medicine, Shanghai Jiao Tong University, Shanghai, China

## Abstract

**Background:**

Systemic lupus erythematosus requires glucocorticoids for management. This study investigates how glucocorticoids influence bone in a SLE mouse model, focusing on bone mineral density (BMD), microstructure, and remodeling markers.

**Methods:**

MRL/lpr and C57BL/6 mice were administered dexamethasone or saline as a control for 4-weeks. Bone assessments included analyses of BMD, bone structure, and serum levels of RANKL and OPG.

**Results:**

Dexamethasone decreased BMD and altered cortical and trabecular bone thickness in both MRL/lpr and C57BL/6 mice. In C57BL/6 mice, cortical bone exhibited increased catabolism while trabecular bone showed signs of increased anabolism, whereas MRL/lpr mice did not show significant changes in bone turnover. Both strains experienced weight loss, with a significant decrease in femur length observed only in C57BL/6 mice. Dexamethasone exacerbated BMD reduction in MRL/lpr mice and halted its increase in C57BL/6 mice. C57BL/6 mice exhibited notable changes in cortical and trabecular bone structure, while MRL/lpr mice didn’t. After receiving dexamethasone, both strains showed higher serum RANKL levels, especially in C57BL/6 mice. OPG decreased in both strains.

**Conclusion:**

Both glucocorticoids and SLE contribute to abnormal bone remodeling through RANKL/OPG pathway.

**Impact:**

- Glucocorticoid (GC) treatment in a mouse model of systemic lupus erythematosus (SLE) leads to significant changes in bone parameters, including decreased bone mineral density (BMD) and alterations in bone structure. Those change are associated with the modulation of RANKL and OPG expression.
- Both GC and inflammation in SLE contribute to BMD reduction, and GC may have a certain protective effect on bone in the early stage of chronic inflammation.
- GC can upregulate RANKL expression and downregulate OPG expression in vivo. During a state of chronic inflammation, RANKL expression increases. However, OPG may not exert a significant influence on inflammatory stimulation.

## Indroduction

Systemic lupus erythematosus (SLE) is an autoimmune disease affecting multiple systems, characterized by a high disability rate and mortality. Osteoporosis is a prevalent complication of SLE.^1^ The dynamic equilibrium biological process of bone remodeling involves the interaction between osteoblasts and osteoclasts. When this balance is disrupted, leading to bone resorption surpassing bone formation, conditions such as bone loss and osteoporosis occur.^2^ Various chronic diseases, including type 1 diabetes mellitus,^3^ chronic kidney disease,^4^ and many rheumatic diseases^1,5^ impact bone health through degeneration of bone remodeling. Glucocorticoids (GC) can contribute to bone deterioration by suppressing bone formation and osteoclast activity. Paradoxically, GC may also help reduce bone loss through its anti-inflammatory effect counteracting the negative impact on bone.^6^ Therefore, additional markers of bone remodeling are essential.The bioactive proteins Receptor Activator of Nuclear Factor-κB Ligand (RANKL) and Osteoprotegerin (OPG) play a crucial role in regulating the balance of bone metabolism. RANKL binds to RANK on myeloid lineage cells, leading to the differentiation and activation of osteoclast precursors, resulting in increased bone resorption. OPG, a soluble decoy receptor for RANKL, prevents RANKL-RANK binding, inhibiting osteoclast maturation. The RANKL/OPG ratio is pivotal in the bone remodeling process and can be influenced by various cytokines, hormones, and growth factor.^7^ Our prior investigations have verified elevated serum RANKL expression, reduced OPG expression, and decreased vitamin D levels in children experiencing remission from SLE.^8^ To gain deeper insights into the alterations in bone and related bone metabolism indices within the pathological context of SLE, further investigation is warranted. In this study, the MRL/lpr lupus mouse model and control C57BL/6 mice were utilized, with intraperitoneal injection of DEX. Changes in systemic bone mineral density (BMD), femur BMD, bone volume/total volume (BV/TV), trabecular thickness (Tb.Th), cortical thickness (Ct.Th), and serum RANKL and OPG expression after DEX injection were observed using ELISA, dual-energy X-ray absorptiometry (DXA), and micro-computed tomography (MicroCT). The aim was to elucidate the effects of GC on both SLE and normal bone changes.

## Methods

In this study, 16 MRL/lpr lupus mouse models and 16 control C57BL/6 female mice were utilized, all procured from the Shanghai Laboratory Animal Center of the Chinese Academy of Sciences/Shanghai Lacke Laboratory Animal Co., LTD. The studies involving experimentation on animals was reviewed and approved by Institutional Animal Care and Use Committee of Shanghai Children’s Hospital (SHCH-IACUC-2021-XMSB-56). After a minimum adaptation period of 1 week in the animal facility, the mice were divided into four groups. The experiment commenced when the mice reached 12 weeks of age. The body weight of MRL/lpr mice was approximately 30 g, and hormone therapy involved intraperitoneal injection of dexamethasone at 1 mg/kg/day. The control group received intraperitoneal injection of normal saline with an equivalent volume. The entire experimental period spanned 4 weeks. The four groups were designated as the SLE group (SLE), SLE with hormone therapy group (SLE + DEX group), control group (CTL), and hormone control group (normal mouse hormone injection), each consisting of 8 mice. Weekly weight measurements were systematically recorded.

BMD of living mice in each group were assessed using a small animal DXA (Small animal dual-energy X-ray analyzer, Korea OSTEOSYS CO., LTD) at the conclusion of the 4-week experiment. Afterward, the specimens were euthanized and preserved. The specific operational procedure is outlined as follows: (1) mice were intraperitoneally injected with 1% sodium pentobarbital anesthesia (0.1 mg/g) to ensure complete anesthesia; subsequently, DXA testing was conducted. A blood sample of 0.2 ml was obtained from the angular vein using a capillary tube. After centrifugation at 4000 RPM for 5 min, the upper serum was collected and stored at −80 °C in a cryogenic refrigerator. (2) Following blood sample collection, mice were humanely euthanized using cervical dislocation. The abdominal cavity of the euthanized mice was opened layer by layer, and both femurs were completely extracted. The right femur was enveloped in PBS-soaked gauze and stored in a −20 °C refrigerator, while the left femur was fixed in a 4% paraformaldehyde solution.

### Experimental detection and other values

The bone mineral density of mice in each group was assessed using a small animal DXA detector before blood collection. The detector was preheated for half an hour. After ensuring the mice were fully anesthetized and properly positioned at the center of the instrument, the door was closed during the test to read data, with a scanning time of 25 s, requiring the mice to remain under anesthesia throughout.

The bone structure was evaluated using a microCT (SkyScan-1176 Micro CT Imaging system Bruker, Belgium). The left femurs were preserved in alcohol, and after being removed and wiped, they underwent scanning using the SkyScan-1176 MicroCT Imaging system (Bruker, Belgium) with the following parameters: a pixel size of 9 microns, medium resolution, 70 kVp, 114 mu, 0.5 mm AL filtering, a threshold of 120~255 mg HA/CCM, and an integration time of 500 ms. A Region of Interest (ROI) was defined as 10% of the total length of the femur under the distal growth plate, representing trabecular bone. Another ROI was set for the area with a central height of 0.3 mm of the femur, representing cortical bone. Measurements included bone trabecular BMD, Tb.Th, bone cortical BMD, Ct.Th, and BV/TV.

Serum RANKL and OPG were detected by Mouse TNFSF11/RANKL(Tumor necrosis factor ligand superfamily member 11) ELISA Kit and Mouse OPG(Osteoprotegerin) ELISA Kit (Zeye Biotech, Shanghai, China).

### Statistical analysis

SPSS 25.0 statistical software package was used for statistics, and all measurement data were represented by mean ± standard deviation. Comparison between the mean of two samples was performed by t test, and *P* < 0.05 was considered statistically significant.

## Results

### Effects of GC on body weight of mice

**Fig. 1:**
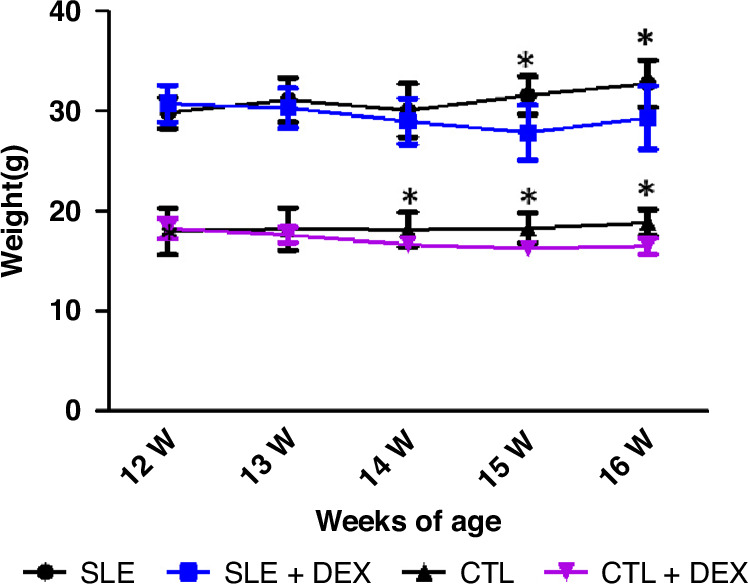
Alterations in the weight of MRL/LPR (SLE) and C57BL/6 (CTL) mice following DEX injection (*P < 0.05). The weight growth trajectory of mice in the SLE and CTL groups, aged 12–16 weeks, after 4 weeks of DEX injection, was compared with the control group. The weight growth pattern in both groups was suppressed by DEX, with a more pronounced decline observed with increasing injection duration. Upon comparing the weight of MRL/LPR (SLE) and C57BL/6 (CTL) mice after DEX injection, the changes became more apparent with prolonged injection time. In the CTL + DEX group, the weight was significantly lower than that in the CTL group after 1 week of injection (beyond 13 weeks of age), and in the SLE + DEX group, the weight was lower than that in the SLE group at 15w and 16w (*P < 0.05, *n* = 8).

MRL/lpr mice can survive up to 16 weeks without experiencing spontaneous tumors or death. To observe the impact of DEX on the weight development of mice, we recorded the weights of mice from 12 to 16 weeks after DEX injection (Table 1a, b, Fig. 1). The results revealed a general trend of weight increase in the untreated groups. However, in the DEX injection group (SLE + DEX), the growth trend of body weight was noticeably restrained, and a more pronounced trend of weight loss was observed. By 15 and 16 weeks, the weight of the SLE + DEX group was significantly lower than that of the SLE group (*P* < 0.05). A similar trend was observed in the C57BL/6 mice, where the weight loss following DEX injection was more pronounced compared to MRL/lpr mice. Notably, the weight of the injection group (CTL + DEX) began to decrease significantly compared to the non-injection group (CTL) starting from 1 week after injection (13 weeks). These results clearly indicate a general trend of weight increase in mice not receiving steroids, whereas the mice receiving DEX experienced weight loss.

**Table 1:** a The weight changes in MRL/lpr mice following DEX injection (*P < 0.05, *n* = 8). b The weight changes in C57BL/6 mice following DEX injection (*P < 0.05, *n* = 8).

A				
Weeks of age	SLE(g)	SLE + DEX(g)	*t*	*P*
12w	29.84 ± 1.55	30.75 ± 1.86	0.9978	0.3353
13w	31.14 ± 2.22	30.31 ± 1.96	0.7367	0.4735
14w	30.08 ± 2.67	28.99 ± 2.29	0.8184	0.4268
15w	31.58 ± 1.91	27.85 ± 2.74	2.949	0.0106^*^
16w	32.79 ± 2.35	29.6 ± 3.3	2.311	0.0365^*^

B				
Weeks of age	CTL(g)	CTL + DEX(g)	*t*	*P*
12w	17.98 ± 2.33	18.2 ± 1.02	1.143	0.2720
13w	18.2 ± 2.12	17.6 ± 0.81	2.913	0.0114^*^
14w	18.16 ± 1.73	16.7 ± 0.72	5.248	0.0001^*^
15w	18.3 ± 1.48	16.3 ± 0.68	6.235	<0.0001^*^
16w	18.84 ± 1.34	16.5 ± 0.8	6.463	<0.0001^*^

**Fig. 2:**
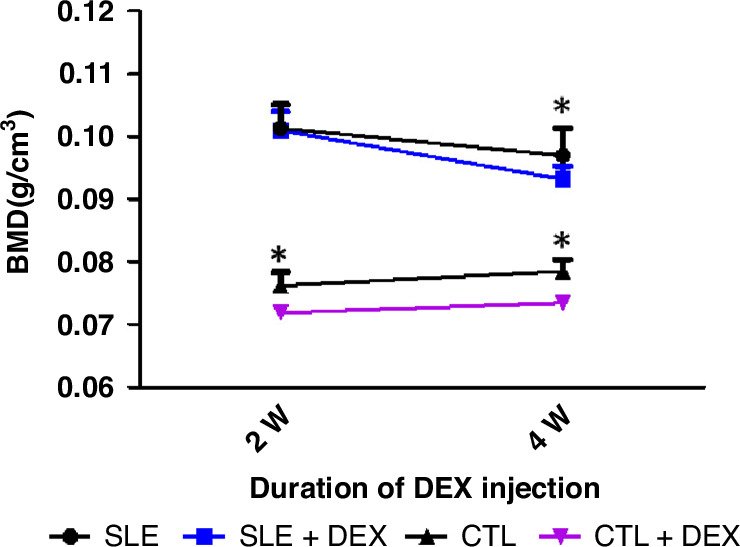
Alterations in bone mineral density (BMD) were observed in MRL/LPR (SLE) and C57BL/6 (CTL) mice following DEX injection (*P < 0.05). The BMD trend lines of the SLE group and CTL group were compared with their respective control groups at 2 weeks (14 age weeks) and 4 weeks (16 age weeks) after Dex injection. The BMD of SLE mice exhibited a decreasing trend, with a more noticeable decline after Dex injection, while the BMD of CTL mice was inhibited post-Dex injection. The comparison of BMD between MRL/LPR (SLE) and C57BL/6 (CTL) mice after Dex injection revealed that the BMD of the CTL+Dex group was significantly lower than that of the CTL group after 2 and 4 weeks of Dex injection. The BMD of the SLE+Dex group was lower than that of the SLE group after 4 weeks of Dex injection (*P < 0.05, *n* = 8).

*Effect of GC on bone mass in mice* (Table 2a, b, Fig. 2) Both chronic inflammation and GC stimulate RANKL expression, but OPG expression may not be as sensitive to inflammation. The decrease in bone mineral density resulting from GC treatment and the significant increase in serological RANKL/OPG suggest a potential correlation

**Table 2:** a BMD comparison in MRL/lpr mice following DEX injection (*P < 0.05, *n* = 8). b BMD comparison in C57BL/6 mice following DEX injection (*P < 0.05, *n* = 8).

A				
BMD	SLE(g/cm^3^)	SLE + DEX(g/cm^3^)	*t*	*P*
2W	0.101 ± 0.00379	0.101 ± 0.00300	1.853	0.0851
4W	0.0969 ± 0.00434	0.0931 ± 0.00213	2.202	0.0449^*^
t	2.145	5.984	–	–
P	0.05^*^	0.0001^*^	–	–

B				
BMD	CTL(g/cm^3^)	CTL + DEX(g/cm^3^)	*t*	*P*
2W	0.0762 ± 0.00217	0.0718 ± 0.00277	3.498	0.0035^*^
4W	0.0785 ± 0.00194	0.0734 ± 0.00257	4.421	0.0006^*^
t	2.246	1.209	–	–
P	0.0413^*^	0.2468	–	–

The BMD comparison between the MRL/lpr mice (SLE group) without DEX treatment revealed a decrease in BMD, while the C57BL/6 mice (CTL group) exhibited increased BMD, and the difference was statistically significant (*P* < 0.05). Following DEX injection, there was a more significant decrease in BMD in MRL/lpr mice (SLE + DEX group). The upward trend of BMD in C57BL/6 mice (CTL + DEX group) was hindered. Comparison at 2 weeks after DEX injection (14 weeks of mouse age) showed no significant difference in BMD between the SLE + DEX group and the SLE group. However, BMD in the CTL + DEX group was significantly lower than that in the CTL group, with the difference being statistically significant (*P* < 0.05). After 4 weeks of DEX injection, BMD in both SLE + DEX and CTL + DEX groups was lower than that in the SLE and CTL groups, and the difference was statistically significant (*P* < 0.05).

**Table 3:** a Comparison of the femur length of MRL/lpr mice following DEX injection (*n* = 8). b Comparison of the femur length of C57BL/6 mice following DEX injection (*P < 0.05, *n* = 8)

A				
Length	SLE(cm)	SLE + DEX(cm)	*t*	*P*
4 W	16.16 ± 0.34	16.1 ± 0.25	0.4066	0.6904

B				
Length	CTL(cm)	CTL + DEX(cm)	*t*	*P*
4 W	14.81 ± 0.49	14.55 ± 0.69	2.202	0.0449^*^

**Fig. 3:**
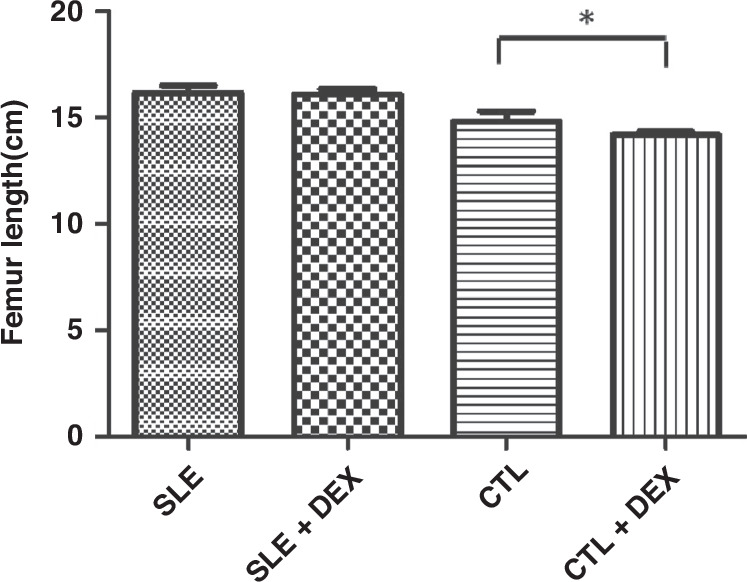
In the MRL/LPR(SLE) mice, the femoral length of the SLE + DEX group showed no significant change compared to the SLE group, the difference was not statistically significant (*P* > 0.05, *n* = 8). Conversely, in the C57BL/6 (CTL) mice, the femur length of the CTL + DEX group was significantly lower than that of the CTL group, with the difference being statistically significant (*P < 0.05, *n* = 8).

### Effects of GC on femur growth (femur length) in mice (Table 3a, b, Fig. 3)

Following 4 weeks of DEX treatment, the femur length in the MRL/lpr lupus mice (SLE + DEX) group exhibited no significant change compared to the SLE group, with the difference not statistically significant (*P* < 0.05). In C57BL/6 mice, the femur length in the CTL + DEX group was lower than that in the CTL group, and this difference was statistically significant (*P* < 0.05).

### BMD of femoral cortical and trabecular bone measured by MicroCT

**Fig. 4:**
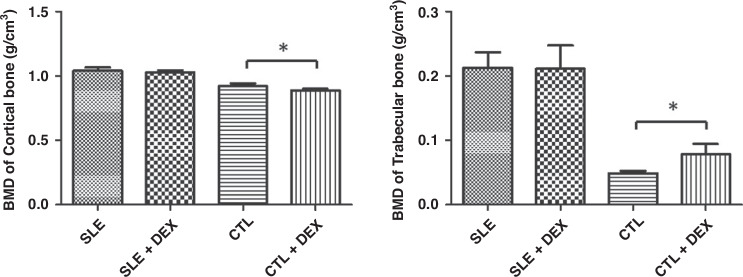
BMD of femoral cortical and trabecular bone was assessed in each group using microCT. In the CTL + DEX group, the BMD of femoral cortical bone was lower than that in the CTL group. Conversely, in trabecular bone, the result was opposite, and both differences were statistically significant (*P < 0.05, *n* = 6). However, there were no significant changes observed in femoral cortical bone and trabecular bone between the SLE group and SLE + DEX group; in both cases, the differences were not statistically significant (*P > 0.05, *n* = 6).

No significant changes were observed in the BMD of femoral cortical bone and trabecular bone when comparing the SLE + DEX group and the SLE group (Table 4a, Fig. 4), and the difference was not statistically significant. However, the BMD of cortical bone in the CTL + DEX group was lower than that in the CTL group, while in trabecular bone, the trend was opposite (Table 4b, Fig. 4). These differences were found to be statistically significant (*P* < 0.05).

**Table 4:** a Comparison of femur BMD of MRL/lpr mice following DEX injection (*n* = 6). b Comparison of femur BMD of C57BL/6 mice following DEX injection (*P < 0.05, *n* = 6).

A				
BMD	SLE(g/cm^3^)	SLE + DEX(g/cm^3^)	*t*	*P*
cortical bone	1.041 ± 0.0267	1.028 ± 0.0160	0.9629	0.3583
trabecular bone	0.213 ± 0.0242	0.211 ± 0.0363	0.08295	0.9355

B				
BMD	CTL(g/cm^3^)	CTL + DEX(g/cm^3^)	*t*	*P*
cortical bone	0.921 ± 0.0225	0.888 ± 0.0132	3.108	0.0111*
trabecular bone	0.0483 ± 0.00410	0.0785 ± 0.0160	4.478	0.0012*

### The thickness of femoral cortical bone and trabecular bone of mice

**Fig. 5:**
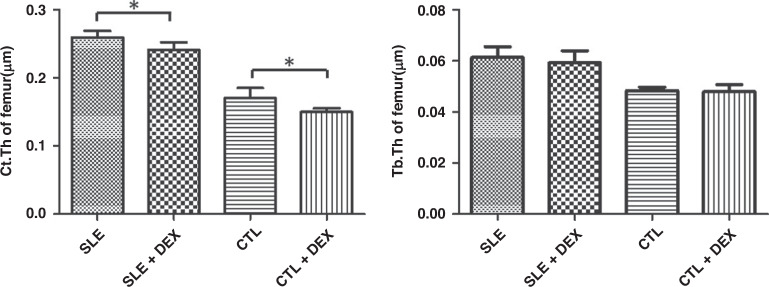
Thickness of femoral cortical bone and trabecular bone was assessed in each group using microCT. The femoral cortical bone thickness in both the SLE + DEX and CTL + DEX groups was significantly lower than that in their respective SLE and CTL groups, and both differences were statistically significant (*P < 0.05, *n* = 6). However, there was no observable change in trabecular bone thickness between the SLE + DEX group and the CTL + DEX group when compared with their corresponding SLE and CTL groups, and in both cases, the differences were not statistically significant (*P > 0.05, *n* = 6).

**Fig. 6:**
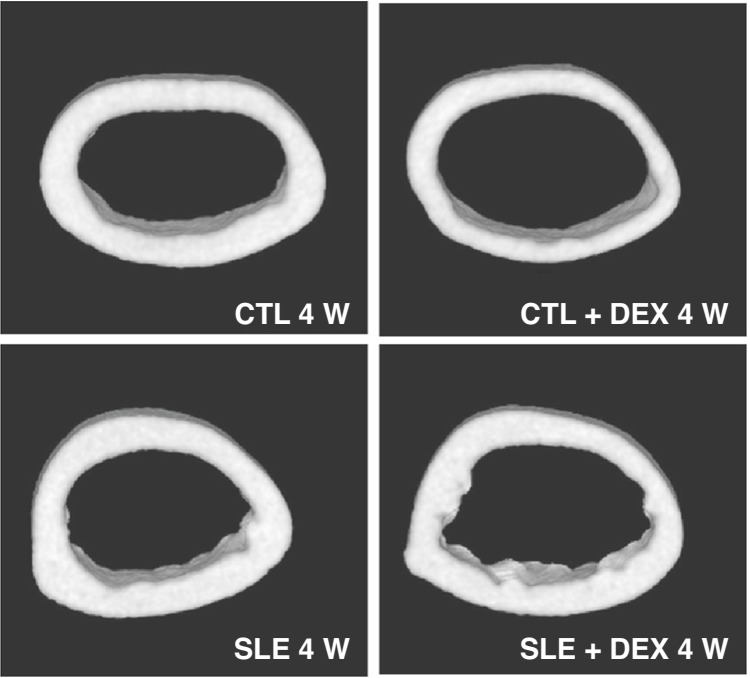
3D MicroCT imaging of femoral cortical bone. In the MRL/LPR mice, the surface of the medullary cavity of the bone cortex was irregular. The femoral cortex of the SLE + DEX group was thinner than that of the SLE group, and this difference was more pronounced in both the CTL + DEX group and the CTL group, representing the C57BL/6 mice.

Regarding femoral cortical bone thickness, both the SLE + DEX group and CTL + DEX group exhibited significantly lower values compared to the SLE group and CTL group (Table 5a, Fig. 5), and these differences were found to be statistically significant (*P* < 0.05). The thinning of the femoral cortex was more pronounced in C57BL/6 mice following DEX injection. However, in terms of trabecular bone thickness, there were no significant changes observed in the SLE + DEX group and CTL + DEX group when compared with the SLE group and CTL group (Table 5b, Fig. 5). Additionally, the cortical medulla surface of the femoral cortex in MRL/lpr mice was found to be uneven, and the femoral cortex was thinner in the SLE + DEX group compared to the SLE group, with this change being more pronounced in C57BL/6 mice (Fig. 6).

**Table 5:** a Comparison of femur thickness of MRL/lpr mice after DEX injection (*P < 0.05, *n* = 6). b Comparison of femur thickness of C57BL/6 mice after DEX injection (*P < 0.05, *n* = 6).

A				
	SLE(μm)	SLE + DEX(μm)	*t*	*P*
Ct.Th	0.259 ± 0.00986	0.241 ± 0.0114	2.938	0.0148*
Tb.Th	0.0613 ± 0.00422	0.0593 ± 0.00467	0.7976	0.4436

B				
	CTL(μm)	CTL + DEX(μm)	*t*	*P*
Ct.Th	0.170 ± 0.0145	0.150 ± 0.00522	3.311	0.0079*
Tb.Th	0.0483 ± 0.00135	0.0478 ± 0.0029	0.355	0.7299

### Femur cortical and trabecular BV/TV in mice

**Fig. 7:**
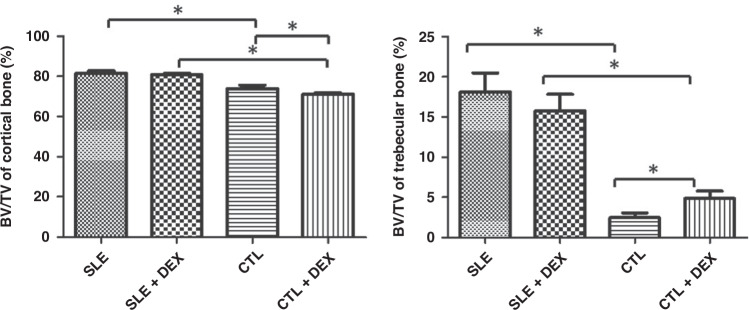
BV/TV assessment of femoral cortical and trabecular bone by microCT. The BV/TV of cortical bone in the CTL + DEX group was significantly lower than that in the CTL group, while the BV/TV of trabecular bone was markedly higher, and these differences were statistically significant. When compared with the SLE group, the BV/TV of SLE + DEX in both cortical and cancellous bone was lower, but the difference was not statistically significant (*P* > 0.05, *n* = 6). The BV/TV of cortical bone and trabecular bone in the SLE + DEX group and SLE group were higher than in the corresponding CTL + DEX group and CTL group, and these differences were statistically significant (*P < 0.05, *n* = 6), with a more pronounced effect observed in trabecular bone. The results suggest that the impact of DEX on the bone cortex of normal mice led to enhanced catabolism, while the effect on trabecular bone was the opposite.

The CTL + DEX group showed a decrease in cortical bone BV/TV compared to the CTL group, while the trabecular bone BV/TV was significantly increased, and these differences were statistically significant (*P* < 0.05). No significant difference was observed in BV/TV between cortical bone and trabecular bone when comparing the SLE + DEX and SLE groups. Additionally, both cortical bone and trabecular bone BV/TV in the SLE + DEX group and SLE group were higher than those in the CTL + DEX group and CTL group, with statistically significant differences (*P* < 0.05), and this effect was more pronounced in trabecular bone (Table 6, Fig. 7).

**Table 6:** Femur cortical and trabecular BV/TV in each group of mice after DEX injection (*n* = 6).

BV/TV(%)	Cortical bone	Trabecular bone
SLE	81.472 ± 1.198	18.125 ± 2.369
SLE + DEX	80.859 ± 0.588^a^	15.779 ± 2.035^e^
CTL	73.950 ± 1.653^b^	2.496 ± 0.566^f^
CTL + DEX	71.031 ± 0.757^c,d^	4.842 ± 0.941^g,h^

^a^There was no significant difference in BV/TV in cortical bone between SLE + DEX group and SLE group (*t* = 1.28, *P* = 0.2865).

^b^The BV/TV in cortical bone was lower in CTL group than SLE group, and the difference was statistically significant (*t* = 9.026, *P* <0.0001).

^c^The BV/TV in cortical bone of CTL + DEX group was lower than that of CTL group, and the difference was statistically significant (*t* = 3.932, *P* = 0.0028).

^d^BV/TV in cortical bone was higher in SLE + DEX group than in CTL + DEX group, and the difference was statistically significant (*t* = 25.10, *P* <0.0001).

^e^There was no significant change in BV/TV in trabecular bone between SLE + DEX group and SLE group, with no statistical significance (*t* = 1.84, *P* = 0.0955).

^f^BV/TV in trabecular bone was lower in CTL group than SLE group, and the difference was statistically significant (*t* = 15.72, *P* <0.0001).

^g^The BV/TV in trabecular bone of CTL + DEX group was lower than that of CTL group, and the difference was statistically significant (*t* = 5.233, *P* = 0.0004).

^h^BV/TV in trabecular bone was higher in SLE + DEX group than in CTL + DEX group, and the difference was statistically significant (*t* = 11.95, *P* <0.0001).

### Serum RANKL and OPG in mice

In comparison to their respective SLE and CTL groups, both the SLE + DEX and CTL + DEX groups demonstrated a higher serum RANKL concentration, exhibiting a statistically significant difference (*P* < 0.05). Prior to DEX treatment, the serum RANKL concentration in the SLE group was higher than that in the CTL group, with a statistically significant difference (*P* < 0.05). However, post-DEX treatment, the serum RANKL concentration in the SLE + DEX group was comparable to that in the CTL + DEX group, and the difference was not statistically significant. This implies that DEX has a more pronounced effect on stimulating RANKL expression in normal mice than in those with SLE (Table 7).

**Table 7:** Concentration of serum RANKL and OPG in each group of mice (*n* = 6).

	RANKL(pmol/L).	OPG(ng/L).	RANKL/OPG(pmol/ng).
SLE	808.97 ± 65.52	1882.97 ± 105.79	0.43 ± 0.047
SLE + DEX	888.69 ± 33.56^a^	1696.01 ± 86.16^e^	0.53 ± 0.042^i^
CTL	699.92 ± 74^b^	1989.21 ± 96.18^f^	0.35 ± 0.039^j^
CTL + DEX	863.19 ± 36.46^c,d^	1690.73 ± 131.54^g,h^	0.51 ± 0.047^k,l^

^a^The serum RANKL concentration in SLE + DEX group was higher than that in SLE group, and the difference was statistically significant (*t* = 2.653, *P* = 0.0242).

^b^The serum RANKL concentration in CTL group was lower than SLE group, and the difference was statistically significant (*t* = −2.703, *P* = 0.0222).

^c^The serum RANKL concentration was higher in CTL + DEX group and CTL group, and the difference was statistically significant (*t* = 3.56, *P* = 0.0052).

^d^There was no significant difference in serum RANKL concentration between SLE + DEX group and CTL + DEX group (*t* = 1.26, *P* = 0.2361).

^e^Serum OPG concentration in SLE + DEX group was lower than that in SLE group, and the difference was statistically significant (*t* = −3.356, *P* = 0.0073).

^f^There was no significant difference in serum OPG concentration between CTL group and SLE group (*t* = 1.82, *P* = 0.0987).

^g^The serum OPG concentration of CTL + DEX group was higher than that of CTL group, and the difference was statistically significant (*t* = 4.487, *P* = 0.0012).

^h^There was no significant difference in serum OPG concentration between SLE + DEX group and CTL + DEX group (*t* = 0.0822, *P* = 0.9361).

^i^The serum RANKL/OPG ratio in SLE + DEX group was higher than that in SLE group, and the difference was statistically significant (*t* = 3.684, *P* = 0.0042).

^j^The serum RANKL/OPG ratio was lower in CTL group than SLE group, and the difference was statistically significant (*t* = −3.161, *P* = 0.0101).

^k^The serum RANKL/OPG ratio between CTL + DEX group and CTL group was higher, and the difference was statistically significant (*t* = 6.452, *P* < 0.0001).

^l^There was no significant difference in serum RANKL/OPG ratio between SLE + DEX group and CTL + DEX group (*t* = 0.4865, *P* = 0.6371).

**Fig. 8:**
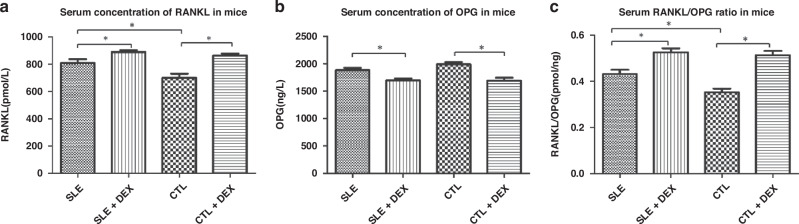
Detection of Serum RANKL and OPG concentrations in mice. **a** Serum RANKL Concentration The detection of serum RANKL concentration in mice revealed higher RANKL levels in the SLE + DEX group and CTL + DEX group compared to their corresponding SLE and CTL groups, and the difference was statistically significant (*P < 0.05, *n* = 6). Before DEX injection, the serum RANKL concentration in the SLE group was higher than in the CTL group, and the difference was statistically significant (*P < 0.05, *n* = 6). However, post-DEX injection, the serum RANKL concentration in the SLE + DEX group was not higher than in the CTL + DEX group, and the difference was not statistically significant (*P < 0.05, *n* = 6). **b** The detection of serum OPG concentration in mice showed a lower OPG concentration in the SLE + DEX group and CTL + DEX group compared to their corresponding SLE and CTL groups, and the difference was statistically significant (*P < 0.05, *n* = 6). **c** The serum RANKL/OPG ratio in each mice group mirrored the pattern observed for serum RANKL.

The serum OPG concentration in both the SLE + DEX and CTL + DEX groups decreased compared to their corresponding SLE and CTL groups, and the differences were statistically significant (*P* < 0.05). There was no significant difference in the serum OPG concentration between the SLE + DEX group and CTL + DEX group. Additionally, there was no difference in the serum OPG concentration between the SLE group and CTL group. The ratio of serum RANKL/OPG aligned with the expression pattern of serum RANKL (Fig. 8).

All the bone structure parameters and serum levels of RANKL and OPG in four groups of mice were presented in Table 8.

**Table 8:** Bone structure parameters and serum RANKL/OPG in each group of mice (*n* = 6).

		SLE	SLE + DEX	CTL	CTL + DEX
Whole body BMD		0.0969 ± 0.00434	0.0931 ± 0.00213	0.0785 ± 0.00194	0.0734 ± 0.00257
Femur length(cm)		16.16 ± 0.34	16.1 ± 0.25	14.81 ± 0.49	14.55 ± 0.69
Femur BMD (g/cm^3^)	Cortical bone	1.041 ± 0.0267	1.028 ± 0.0160	0.921 ± 0.0225	0.888 ± 0.0132
	Trabecular bone	0.213 ± 0.0242	0.211 ± 0.0363	0.0483 ± 0.00410	0.0785 ± 0.0160
Ct.Th(μm)		0.259 ± 0.00986	0.241 ± 0.0114	0.170 ± 0.0145	0.150 ± 0.00522
Tb.Th(μm)		0.0613 ± 0.00422	0.0593 ± 0.00467	0.0483 ± 0.00135	0.0478 ± 0.0029
BV/TV (%)	Cortical bone	81.472 ± 1.198	80.859 ± 0.588	73.950 ± 1.653	71.031 ± 0.757
	Trabecular bone	18.125 ± 2.369	15.779 ± 2.035	2.496 ± 0.566	4.842 ± 0.941
RANKL(pmol/L)		808.97 ± 65.52	888.69 ± 33.56	699.92 ± 74	863.19 ± 36.46
OPG(ng/L)		1882.97 ± 105.79	1696.01 ± 86.16	1989.21 ± 96.18	1690.73 ± 131.54
RANKL/OPG(pmol/ng)		0.43 ± 0.047	0.53 ± 0.042	0.35 ± 0.039	0.51 ± 0.047

## Discussion

Bone remodeling is a dynamic process, intricately balancing bone resorption and formation. Osteoclasts (OC) absorb old bone, while osteoblasts (OB) form new bone. Bone remodeling, a finely tuned equilibrium of bone resorption and formation, relies on the orchestrated interplay between osteoclasts (OC) and osteoblasts (OB). Precise coordination between these cell types is crucial for maintaining equilibrium. OB modulates OC through the OPG/RANKL/RANK signaling system. Systemic Lupus Erythematosus (SLE) frequently leads to osteoporosis, a systemic metabolic bone ailment characterized by diminished bone mass, microstructural deterioration, and heightened bone fragility. The reported incidence of osteoporosis in SLE varies widely, ranging from 1.4 to 68%.^9,10^ The long-term heavy use of Glucocorticoids (GC), a cornerstone in SLE treatment, is a common cause of secondary osteoporosis, inducing osteoporosis by influencing the OPG/RANK/RANKL pathway. Growth failure is common in SLE due to many risk factors including chronic inflammation, malnutrition, and long-term use of corticosteroids.^11^

MRL/lpr mice spontaneously develop a severe form of SLE, characterized by features such as glomerulonephritis, arthritis, and autoantibody production. This model is highly suitable for investigating disease mechanisms and potential therapies. Mice in our study were selected at 12 weeks of age, which corresponds to the juvenile phase in this model. This age is critical as it mirrors the developmental stage of pediatric SLE patients, when both the immune system and bone metabolism are actively evolving. This provided an opportunity to observe the impact of DEX treatment on disease progression and bone health. Our experiments spanned 4 weeks, with assessments conducted at specific intervals (from 12 to 16 weeks). This duration allowed us to evaluate both immediate and longer-term effects of DEX treatment on bone structure and density. Children with SLE experience growth delays more severe than those with other rheumatic immune diseases.^12^ Our comparative study of SLE mice model and C57BL/6 mice revealed that ordinary mice exhibited growth delay one week post-GC injection, whereas lupus mice experienced a three-week lag, indicating a significant weight disparity. Concurrently, femur length in SLE mice showed no significant change after 4 weeks of DEX treatment, while normal mice experienced a significant decrease, suggesting that in the early stages, GC may mitigate disease-induced effects on mouse weight growth by inhibiting inflammation. However, GC exhibited an adverse effect on body weight in the later stages. The delayed growth and development inhibition effect of GC in SLE mice warrants further investigation.

Chronic inflammation and GC jointly inhibit body growth and development, primarily affecting bone development due to increased bone loss. GC, along with other factors, disrupts bone metabolism, shifting the balance from bone formation to resorption, eventually leading to low bone mass and microstructural destruction, culminating in osteoporosis.^13–15^ Studies indicate that the rate of bone loss induced by GC is faster in the early stage but slows in the later stage due to increased apoptosis of osteoblasts and osteocytes, reduced production of RANKL, decreased osteoclast count, and weakened apoptosis and autophagy. The slower bone loss may also result from reduced inflammatory activity and GC dose reduction.^16^ In our study, BMD assessments during and after treatment revealed decreased BMD in SLE model mice at 4 weeks, while control mice showed increased BMD, highlighting the significant impact of SLE on bone mineral density. DEX injection exacerbated BMD reduction in the SLE + DEX group, with a more profound effect than inflammation alone. It suggested that both GC and inflammation in SLE contribute to BMD reduction, with GC potentially having a cumulative effect. However, BMD in SLE mice did not change significantly at 2 weeks post-DEX injection, declining only after 4 weeks, indicating a delayed impact compared to the faster decrease observed in the CTL group. GC may have a certain protective effect on bone in the early stage of chronic inflammation. This time point is about 2-3 weeks after GC treatment in mice and is worthy of further study and exploration in human diseases.

Bone loss rapidly follows GC use, with fracture incidence correlating with cumulative GC dose.^17^ However, studies reveal that, under similar BMD conditions, GC users face a higher risk of osteoporotic fractures than non-users,^18^ suggesting an independent mechanism affecting bone strength through microstructural damage. MicroCT imaging in MRL/LPR mice showed uneven changes in the bone cortex. After DEX injection, the bone cortex of both SLE and control mice became thinner, with the latter exhibiting more pronounced changes. While this mirrored the results of mouse growth and bone mineral density, local BMD of the femur under DEX influence only changed in C57BL/6 mice, with no significant effect on MRL/LPR mice. This disparity may have distinct effects on other bone parts with varying trabecular and dense bone structures, such as the spine. The effect of GC on bone trabeculae with high metabolic activity was more pronounced than on cortical bone, as verified in this study. In C57BL/6 mice, DEX stimulation increased trabecular bone BV/TV but significantly decreased cortical bone BV/TV, indicating enhanced bone catabolism in cortical bone. In SLE mice, the BV/TV of trabecular and dense bone slightly decreased after DEX injection, suggesting a trend of increased catabolism, though statistical significance required larger sample observations. The effects of GC on bone in chronic inflammatory states require further investigation.

RANKL, secreted by preosteoblasts, osteoblasts, and periosteum cells, plays a pivotal role in osteoclast activation. In this study, pathological experiments confirmed evident bone lesions in MRL/LPR mice and GC-treated C57BL/6 mice, characterized by reduced, thinner, and disordered bone trabeculae. TRAP staining revealed activation of osteoclasts in both SLE and GC-treated mice, with enhanced RANKL expression. Both disease and GC played integral roles in osteoclast activation. In addition to the subcortical region, MRL/LPR mice also exhibited osteoclast effects in the bone marrow cavity, and GC reduced TRAP expression in the bone marrow cavity, suggesting differing mechanisms for chronic inflammation and GC in causing bone abnormalities.

The RANKL/OPG ratio represents the negative balance of bone metabolism, with an increased ratio indicating heightened bone absorption and a possible decrease in bone density. GC treatment affects RANKL/OPG differently under various inflammatory states. Ali et al.^19^ found that RANKL, OPG and RANKL/OPG ratio were elevated in SLE patients. In the case of SLE remission, GC is involved in the occurrence and development of abnormal bone remodeling through RANKL/OPG.^8^ In this study, SLE mice exhibited a higher serum RANKL concentration than the CTL group, while OPG showed no significant change, resulting in an increased RANKL/OPG ratio. The observed elevation in RANKL and RANKL/OPG levels during inflammation contrasts with findings reported by Ali et al. Several factors could contribute to this inconsistency, such as differences in experimental models, varying disease stages and severity, sample sizes, statistical power, and potential confounding variables. However, the serum RANKL concentration in both groups after DEX treatment was similar, suggesting a more pronounced effect of DEX on RANKL expression in C57BL/6 mice compared to MRL/LPR mice. In C57BL/6 mice, treatment with DEX resulted in increased RANKL levels and decreased OPG levels, thereby increasing the RANKL/OPG ratio. Consequently, both SLE and C57BL/6 mice exhibited an elevated RANKL/OPG ratio following DEX treatment. Both chronic inflammation and glucocorticoid treatment stimulate RANKL expression, while OPG expression may not respond as sensitively to inflammatory stimuli due to differing regulatory mechanisms. Inflammatory mediators directly upregulate RANKL levels, whereas OPG regulation involves more intricate pathways that are less influenced by inflammation. The significant decrease in BMD observed alongside increased RANKL/OPG ratios suggests a direct correlation between these factors. Elevated RANKL enhances osteoclast activity, leading to heightened bone resorption and reduced BMD. Therefore, a high RANKL/OPG ratio correlates with bone loss, highlighting its importance in assessing bone health under inflammatory and GC-treated conditions. Monitoring changes in the RANKL/OPG ratio can serve as a valuable marker for evaluating bone metabolism and assessing treatment effectiveness in clinical settings. Limitedly, the lack of consistent sample selection prevented correlation analysis in this study, necessitating exploration in future studies.

Sex hormones, particularly estrogen, play a critical role in bone metabolism and structural integrity by regulating the balance between bone resorption and formation through the RANKL/OPG pathway. In females, decreased estrogen levels during menopause or due to hormonal imbalances can lead to increased bone resorption and decreased bone formation, contributing to osteoporosis.^20^ In our study, all experimental mice were female, highlighting the potential influence of sex hormones on bone structure parameters. While our study did not directly measure estrogen levels or their effects, the absence of this variable could have impacted the observed changes in bone structure. Glucocorticoid (GC) treatment, such as with DEX, profoundly affects bone metabolism beyond its direct impact on RANKL/OPG signaling. GCs can disrupt hormonal balance, including estrogen levels, thereby exacerbating bone loss. Future research should further investigate these interactions to better understand their implications for bone health. This includes examining the broader effects of GC treatment on hormonal balance and bone metabolism, and considering the role of sex hormones like estrogen in modulating bone density and structure.

## Conclusion

Dexamethasone treatment led to decreased BMD and alterations in bone structure, with notable differences between MRL/lpr and C57BL/6 mice. GC can stimulate the expression of RANKL and inhibit the expression of OPG in vivo. During a state of chronic inflammation, the expression of RANKL is up-regulated. However, OPG may not exert a significant influence on inflammatory stimulation. Both glucocorticoids and SLE contribute to abnormal bone remodeling via RANKL/OPG pathways.

## Data Availability

Data sharing will be available from W.H. upon a reasonable request.

## References

[1] Ceccarelli, F. et al. Fragility fractures in lupus patients: Associated factors and comparison of four fracture risk assessment tools. *Lupus* **32**, 1320–1327, (2023). 37698854 10.1177/09612033231202701

[2] Ramesh, P., Jagadeesan, R., Sekaran, S., Dhanasekaran, A. & Vimalraj, S. Flavonoids: Classification, Function, and Molecular Mechanisms Involved in Bone Remodelling. *Front. Endocrinol.* **12**, 779638 (2021). 10.3389/fendo.2021.779638 PMC8649804 34887836

[3] Qian, W. et al. Bone intrinsic material and compositional properties in postmenopausal women diagnosed with long-term Type-1 diabetes. *Bone* **174**, 116832, (2023). 37385427 10.1016/j.bone.2023.116832 PMC11302406

[4] Aguilar, A. et al. Pathophysiology of bone disease in chronic kidney disease: from basics to renal osteodystrophy and osteoporosis. *Front. Physiol.* **14**, 1177829, (2023). 37342799 10.3389/fphys.2023.1177829 PMC10277623

[5] Tong, J. J. et al. Prevalence and risk factors associated with vertebral osteoporotic fractures in patients with rheumatoid arthritis. *Clin. Rheumatol.* **39**, 357–364, (2020). 31691041 10.1007/s10067-019-04787-9

[6] Corrado, A. et al. Influence of glucocorticoid treatment on trabecular bone score and bone remodeling regulators in early rheumatoid arthritis. *Arthritis. Res. Ther.* **23**, 180, (2021). 34229744 10.1186/s13075-021-02562-3 PMC8261978

[7] Xue, J. Y., Ikegawa, S. & Guo, L. Genetic disorders associated with the RANKL/OPG/RANK pathway. *J. Bone Min. Metab.* **39**, 45–53, (2021). 10.1007/s00774-020-01148-4 32940787

[8] Hao, S. et al. Bone remodeling serum markers in children with systemic lupus erythematosus. *Pediatr. Rheumatol. Online J.* **20**, 54, (2022). 35897105 10.1186/s12969-022-00717-3 PMC9327424

[9] Mok, C. C., Mak, A. & Ma, K. M. Bone mineral density in postmenopausal Chinese patients with systemic lupus erythematosus. *Lupus* **14**, 106–112, (2005). 15751814 10.1191/0961203305lu2039oa

[10] Bultink, I. E. Osteoporosis and fractures in systemic lupus erythematosus. *Arthritis. Care Res.* **64**, 2–8, (2012). 10.1002/acr.20568 22213721

[11] Jongvilaikasem, P. & Rianthavorn, P. Longitudinal growth patterns and final height in childhood-onset systemic lupus erythematosus. *Eur. J. Pediatr.* **180**, 1431–1441, (2021). 33389070 10.1007/s00431-020-03910-2

[12] Batu, E. D. Glucocorticoid treatment in juvenile idiopathic arthritis. *Rheumatol. Int.* **39**, 13–27, (2019). 30276425 10.1007/s00296-018-4168-0

[13] Ono, T., Hayashi, M., Sasaki, F. & Nakashima, T. RANKL biology: bone metabolism, the immune system, and beyond. *Inflamm. Regen.* **40**, 2, (2020). 32047573 10.1186/s41232-019-0111-3 PMC7006158

[14] Compston, J. E., McClung, M. R. & Leslie, W. D. Osteoporosis. *Lancet* **393**, 364–376, (2019). 30696576 10.1016/S0140-6736(18)32112-3

[15] Güler-Yüksel, M., Hoes, J. N., Bultink, I. E. M. & Lems, W. F. Glucocorticoids, Inflammation and Bone. *Calcif. Tissue Int.* **102**, 592–606, (2018). 29313071 10.1007/s00223-017-0335-7

[16] Teitelbaum, S. L. Bone: the conundrum of glucocorticoid-induced osteoporosis. *Nat. Rev. Endocrinol.* **8**, 451–452, (2012). 22688626 10.1038/nrendo.2012.89

[17] van Staa, T. P., Leufkens, H. G. & Cooper, C. The epidemiology of corticosteroid-induced osteoporosis: a meta-analysis. *Osteoporos. Int.* **13**, 777–787, (2002). 12378366 10.1007/s001980200108

[18] Van Staa, T. P. et al. Bone density threshold and other predictors of vertebral fracture in patients receiving oral glucocorticoid therapy. *Arthritis Rheum.* **48**, 3224–3229, (2003). 14613287 10.1002/art.11283

[19] Ali, R. et al. Serum RANKL, osteoprotegerin (OPG) and RANKL/OPG ratio in children with systemic lupus erythematosus. *Lupus* **28**, 1233–1242, (2019). 31403902 10.1177/0961203319867129

[20] Ma, C. et al. Dihydrotanshinone I attenuates estrogen-deficiency bone loss through RANKL-stimulated NF-κB, ERK and NFATc1 signaling pathways. *Int. Immunopharmacol.* **123**, 110572, (2023). 37572501 10.1016/j.intimp.2023.110572

